# Quality of life of breast cancer survivors: a comparison of breast conserving surgery versus total mastectomy with and without immediate reconstruction: a prospective cohort study

**DOI:** 10.1097/MS9.0000000000000607

**Published:** 2023-04-13

**Authors:** Lubna M. Vohra, Saad Malik Javed, Dua Jabeen, Syeda Sakina Abidi, Muhammad Umair Tahseen

**Affiliations:** Departments of a Breast Surgery; b Surgery, Aga Khan University Hospital; c Department of Medical Oncology, Jinnah Postgraduate Medical Centre; d Department of Surgery, Dow University of Health Sciences, Karachi, Pakistan

**Keywords:** breast conserving surgery (BCS), breast reconstruction, breast, mastectomy, quality of life

## Abstract

Quality of life after breast cancer surgery is frequently ignored during and after treatment in many cancer survivors. To enhance this aspect of patient’s life should be the primary goal of every cancer treatment. Therefore, the present study aimed to highlight the quality of life and patients’ satisfaction with their breast cosmesis following breast conserving surgery (BCS), total mastectomy with and without reconstruction.

**Material and methods:**

Data were collected prospectively from cancer patients who had undergone breast surgery at our institution from 1 January 2015 to 31 December 2021. The validated Breast-Q questionnaires were utilized for conducting patient interviews and mean scores between three cohorts were compared using one-way ANOVA test / Kruskal–Wallis test.

**Results:**

Overall, 210 patients were recruited in which 70 patients (33.3%) had undergone BCS, 71 patients (33.8%) had total mastectomy only and 69 (32.9%) patients had total mastectomy with reconstruction. Physical well-being scores were consistent between the three groups while patients operated with total mastectomy with reconstructive surgery scored higher in sexual and psychosocial health measures as compared to patients of total mastectomy. However, BCS patients were the most satisfied with their cosmetic outcome following patients of total mastectomy with reconstruction and without reconstruction.

**Conclusion:**

Reconstruction postmastectomy has a positive impact on sexual and psychosocial well-being of survivors; however, those who had breast conservation were more satisfied with cosmetic outcome post-surgery as compared with mastectomy with or without reconstruction.

## Introduction

Highlights

- Quality of life should be considered primarily while planning treatment to maintain patients’ self-esteem, sexual life and cosmesis.
- Patients who underwent postmastectomy immediate reconstruction had a better quality of life as compared with mastectomy alone.
- Satisfaction with cosmetic outcome of breasts found improved in patients with breast conservation versus mastectomy with or without reconstruction.

About one in seven women in lifetime suffered from breast cancer making it the most common invasive cancer in this sex worldwide with higher incidence in developed world^1^,^2^. In a low middle income country like Pakistan, it accounts for the most frequently diagnosed and leading cause of cancer deaths in female population having an estimated 5-year prevalence between 34 038 and 90 000 newly diagnosed cases and 16 232 deaths in 2017 which is an alarming situation^3^. Therefore, newer innovative treatment strategies are continuously being introduced with the primary aim of improving quality of life and patient’s satisfaction with their cosmetic outcome following breast surgery.

Most early-stage breast cancers (T1 and T2 cancers with or without nodal involvement) are amenable to breast conserving surgery (BCS) followed by radiotherapy^4^. Nevertheless, in some cases mastectomy appears to be the treatment of choice. These cases include multi-centric tumours with high probability of recurrence, T4 disease or BRCA cases or carriers, pregnant woman, collagen vascular disease or with a prior history of irradiation, anticipated poor cosmesis and most importantly patient’s preference^5^–^7^.

Many prospective, randomized trials (National Cancer Institute, Milan Cancer Institute) have demonstrated equivocal survival benefit after BCS versus mastectomy^4^,^8^. Certainly, BCS usually preserves femininity, self-esteem and confidence and thus associated with reduced psychological and sexuality disturbance making it the most favourable treatment option for female patients^9^. On the contrary, some studies had also reported that females who received BCS were more likely to suffer from fear of local recurrence than mastectomy which disturbed their routine life with psychological distress^10^. This indicates that patients usually take into account the long-term consequences of various approaches of surgery on their quality of life and physical appearance when making decision at time of surgery.

Breast Reconstruction either immediate or delayed is essentially a solution for those females who would experience psychosocial and disfigurement issues after ablative surgery. This fact has been proved by the study of Wellisch *et al*.^11^ which showed that patients undergoing breast reconstruction were more stable psychologically and satisfied with their cosmetic results. A study conducted by Howes and colleagues on quality of life had revealed that women with breast reconstruction following mastectomy were living a better life and were more contented with their outlook than patients with total mastectomy and BCS. Moreover, in this study the physical well-being of BCS patients was unsatisfactory and so is the sexual well-being of mastectomy patients^12^. Relevantly, another study by Jagsi *et al*.^13^ also concluded that there was no difference in cosmetic satisfaction between patients with breast conservation and mastectomy with reconstructive surgery.

Paucity of research exists on quality of life and patient’s satisfaction with the cosmetic outcome following different operative interventions on breasts in Pakistan which report various psychosocial and physical challenges such as anxiety, depression and fatigue faced by the patients. To the best of our knowledge there are no studies addressing this crucial matter. Therefore, to attenuate gaps in knowledge present study is designed to unravel patients’ data on quality of life and their satisfaction with the cosmesis following BCS, total mastectomy with and without reconstruction. We hypothesized that patients who underwent breast reconstruction were more satisfied psychologically, sexually, and happy with their outlook than those who underwent mastectomy. We also speculated that BCS patients scored higher in physical health as compared with other two intervention groups.

## Material and methods

This prospective, cohort study was conducted among female patients who underwent BCS, total mastectomy with and without reconstruction at Breast Surgery Unit of our tertiary care centre from 1 January 2015 to 31 December 2021. The research project was ethically approved by the Research Ethics Committee of the Institution and principles of Helsinki Declaration were duly followed^14^. The study has been registered with German Clinical Trials Register (DRKS) and carried out according to the reporting guidelines of STROCSS criteria^15^.

Information on clinical and treatment characteristics of all three patient’s group was taken from the medical records on a self-designed form by the researcher. Data on quality of life and satisfaction with cosmetic outcome among breast surgery patients was assessed using validated Breast-Q questionnaire version 2.0 developed by Memorial Sloan-Kettering Cancer Center and the University of British Columbia. Its first issue was published in 2009 and since then, it has been utilized by the health personnels worldwide to evaluate outcome measures among patients after different surgical interventions. Numerous studies had identified it as a significant tool for this purpose and its development has obliterated the use of multiple questionnaires with limited authenticity. Reliability of this questionnaire has been supported by high Cronbach’s α coefficients (≥0.78) and item-total correlations (range of means, 0.65–0.91). Validity is supported by inter-scale correlations, convergent and divergent hypotheses. License to use Breast-Q has been acquired and the scales have been translated in Urdu language in accordance and after approval from MAPI trust (https://mapi-trust.org/questionnaires/) and the above two mentioned institutes^16^,^17^. However, Breast-Q–Breast cancer core scale (Postoperative version) for BCS evaluate only satisfaction with breasts and physical well-being in patients, sexual and psychosocial is not applicable to BCS group. Patients were interviewed either during routine follow-up visits or were called back for an interview if their visit to the hospital was not scheduled. Sociodemographic characteristics of patients were also asked during the interview and included. Written and informed consent was obtained from all patients prior to the interviews, and they were assured that their identity was kept anonymous to any other person other than the researchers. A healthcare worker not directly involved with care of these patients conducted the interview; this was either an oncology nurse or a doctor. Patients who had died, those who had undergone breast reconstruction following BCS and were unwilling to participate or refused to give informed consent were excluded from the study.

Data was entered using Breast-Q software. The software automatically transformed raw collected data to summary scores ranging from 0 (very dissatisfied) to 100 (very satisfied) for each scale. For all Breast-Q scales, a higher score denotes greater satisfaction or better quality of life. A mean change of 5–10 on a multi-item scale is perceived as “a little” change, 10–20 as “a moderate” change, and greater than 20 as “a maximal” change. Breast-Q is a specifically designed tool to assess patient reported outcomes in terms of quality of life and satisfaction of patients from their point of view, who undergo surgical procedures for treatment of breast cancer. Different scales for mastectomy, reconstruction and conservation consist of five to nine scales with three to five items in each scale. Scores from each scale range from 0 (very dissatisfied) to 100 (very satisfied).

Data were analyzed on statistical package for the social sciences (SPSS) (version 23.0). The quantitative variables were reported as means and standard deviation/median (interquartile range) and the categorical variables were represented by frequencies and percentages. The categorical variables were assessed by χ^2^/ Fisher Exact test. A one-way ANOVA test / Kruskal–Wallis test was used to compare mean Breast-Q scores and assessment of satisfaction with cosmesis between three groups. A *P* value of less than 0.05 was considered significant.

## Results

Baseline characteristics of the study population are described in Table 1. A total of 210 patients were assessed through Breast-Q in this study, out of them 70 patients (33.3%) had undergone BCS, 71 patients (33.8%) had total mastectomy without reconstruction and 69 (32.9%) patients had total mastectomy with reconstruction. The age of the most participants at diagnosis were range of 46–55 years with 44.2% for BCS, 43.6% for total mastectomy without reconstruction and 46.4% for total mastectomy with reconstruction. A large proportion of patients were house makers in all three cohorts (mean= 88.6%) and substantial number of them had a household income between 20 000 and 50 000 PKR (mean= 55.2%).

**Table 1:** Sociodemographic features of women with breast cancer

Characteristics	Breast conserving surgery (*n*=70), *n* (%)	Total mastectomy without reconstruction (*n*=71), *n* (%)	Total mastectomy with reconstruction (*n*=69), *n* (%)	*P*
Age at diagnosis				
<46 years	21 (30)	18 (25.3)	29 (42)	0.045*
46–55 years	31 (44.2)	31 (43.6)	32 (46.4)	
>56 years	18 (25.7)	22 (31)	8 (11.6)	
Occupation				
Employed	8 (11.4)	9 (12.7)	7 (10.1)	0.701*
Unemployed	62 (88.6)	62 (87.3)	62 (89.9)	
Marital status				
Married	61 (87.2)	67 (94.3)	60 (87)	0.077*
Single	5 (7.1)	2 (2.8)	8 (11.6)	
Widow	4 (5.7)	2 (2.8)	0 (0)	
Divorce	0	0	1 (1.4)	
Education				
Illiterate	9 (12.9)	16 (22.5)	2 (2.9)	0.012*
Intermediate	31 (44.2)	31 (43.7)	37 (53.6)	
Bachelors	29 (41.4)	23 (32.4)	30 (43.5)	
Master or higher	1 (1.4)	1 (1.4)	0	
Household income				
<20 000	2 (2.9)	1 (1.4)	1 (1.4)	0.793*
20 000–50 000	36 (51.4)	43 (60.6)	37 (53.6)	
50000+	32 (45.7)	27 (38)	31 (45)	
Menopause				
Premenopausal	36 (51.4)	30 (42.3)	42 (60.9)	*P*=0.073*
Postmenopausal	34 (48.6)	41 (57.7)	27 (39.1)	

* Fisher exact test.

**Figure 1:**
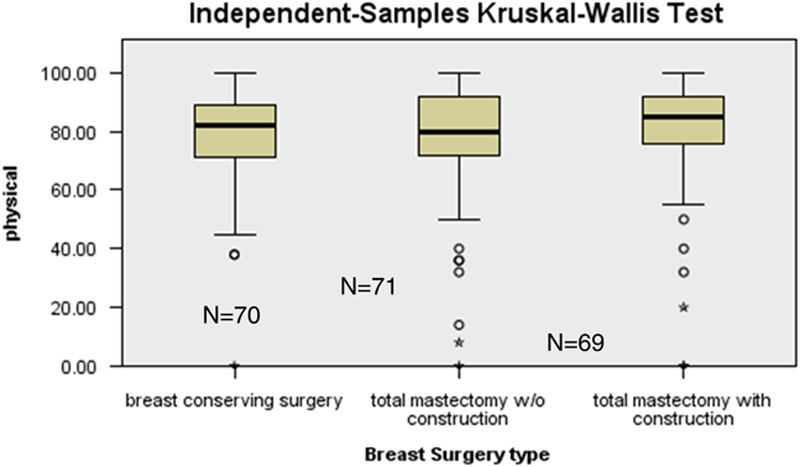
Distribution of Physical Well-being Breast-Q scores among types of breast surgery.

**Figure 2:**
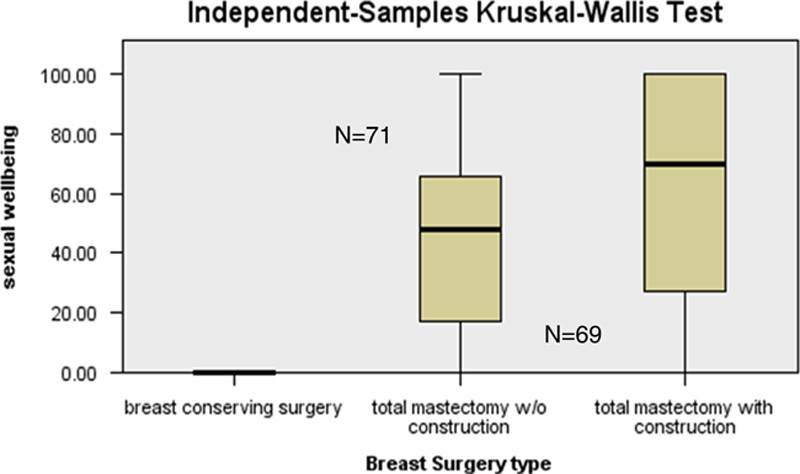
Distribution of Sexual Well-being Breast-Q scores among types of breast surgery.

**Figure 3:**
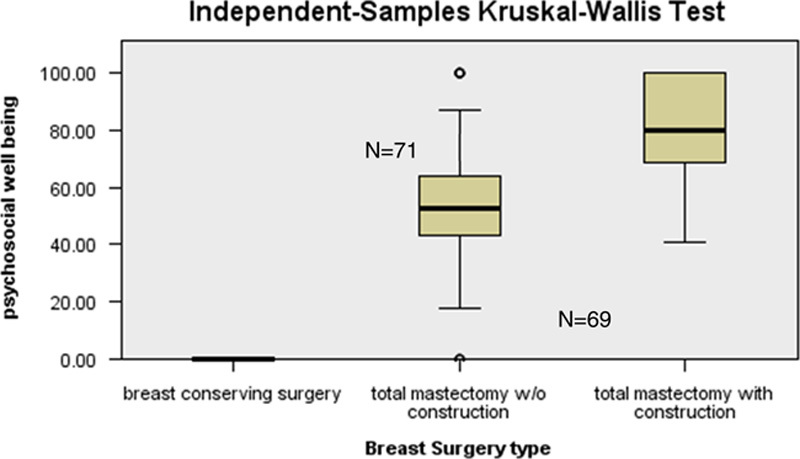
Distribution of Psychosocial Well-being Breast-Q scores among types of breast surgery.

**Figure 4:**
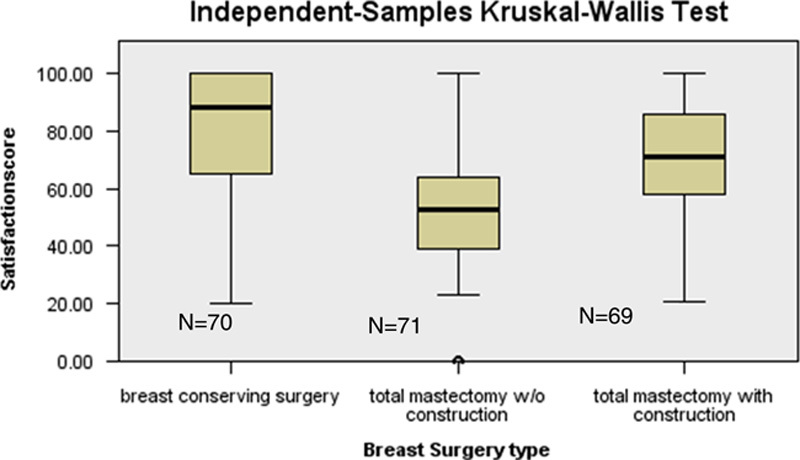
Distribution of satisfaction Breast-Q scores among types of breast surgery.

Results from the Breast-Q questionnaires are described in Figures 1 – 4. Referring to the domain of physical well-being of chest (Fig. 1), there was no significant difference in Breast-Q scores of patients between BCS, total mastectomy without reconstruction and total mastectomy with reconstruction (mean score of 80.0, *P*=0.417).

In terms of the sexual well-being (Fig. 2), patients who underwent total mastectomy with reconstruction had found it more satisfactory than patients who had undergone total mastectomy without reconstruction, respectively, (mean score of 61.0 versus mean score of 45.0 giving a significant *P* value of 0.001). Breast-Q questionnaire is not applicable to assess sexual well-being in BCS group.

In the domain of psychosocial well-being (Fig. 3), Breast-Q scores for patients who underwent total mastectomy without reconstruction (mean score 54.8) were lower than patients of total mastectomy with reconstructive surgery group (mean score 79.9). A statistically significant *P* value was found that is 0.001.

Breast-Q scores for satisfaction with breast (Fig. 4) were quite favourable for patients with BCS (mean score 84.0) than total mastectomy with or without reconstruction giving significant results *P*=0.001. However, patients who were operated with total mastectomy and reconstruction were more satisfied with the outlook of their breasts versus patients of total mastectomy only (mean score 70.0 versus mean score 52.0).

## Discussion

With increasing prevalence of breast cancer in younger population worldwide and improved survival rate women are more conscious about individualized treatment options which may affect their quality of life in future^18^,^19^. Published and unpublished data from Pakistan revealed the fact that mastectomy is a dominant surgical procedure among surgeons. We are a low middle income country, most of the women are home makers with lack of insurance coverage and insufficient financial resources to choose personalized options, patients declined breast conservation as radiotherapy is a part and parcel of treatment with additional financial burden. Many declined reconstruction because of non-affordability, fear of reconstruction failure or complications which may lead to extended cost.

Our data canvassing found that women who underwent BCS or mastectomy with reconstruction were observed to be more appeased with the contour of their breasts comparing with women who had mastectomy with no reconstructive surgery. This is in line to the study conducted by Fung and colleagues which unveiled that patients who received BCS and breast reconstruction following mastectomy were less distraught about their body appearance than those with total mastectomy alone. However, their study utilized body image (BI-7 and BI-5) score to assess satisfaction with breasts and change in their outlook apart from breast-specific Breast-Q questionnaire^20^. Another observational study performed in hospitals of Iran also delineated that BCS group attained the highest level of satisfaction with surgery followed by patients with mastectomy and then mastectomy with reconstructive intervention^21^.

Women who had mastectomy with reconstruction had a better psychosocial outcome comparing to mastectomy without reconstruction. Dean *et al*.^22^ in his study had also witnessed a decrease in postoperative depression in women who had early reconstruction postmastectomy. Albornoz *et al*.^23^ in another study also published similar results where quality of life measures such as satisfaction with breast, psychosocial and sexual well-being were remarkably improved between preoperative and operative phase in women who underwent mastectomy with reconstruction. Another important finding highlighted in our study is that there was no significant difference in physical wellness of chest in all three groups of women who went through BCS and total mastectomy with or without reconstruction although a previously cited study contraindicated our result as they found worst physical well-being of chest in women that underwent (BCS which they concluded that it could be due to adjuvant radiotherapies^12^. A study conducted by Kim *et al*.^24^ in a Korean Hospital employing the questionnaire of EORTC QLQ-C30 and the BR23 module disclosed that patients in the BCS and total mastectomy with immediate reconstruction groups were more physically better in contrast to the total mastectomy group (*P* < 0.05 each).

Preservation of one’s sexuality after breast cancer treatment is an utmost concern among survivors. Despite of its importance, sexual health and function is not often discussed during counselling before, during and after sustaining treatment for cancer. A study by Ganz *et al*.^25^ carried among 763 survivors diagnosed with breast cancer ~6.3 years ago responded that their physical, social and emotional health was stable satisfactorily but sexual activity with their partner was drastically declined when compared with the initial survey that is from 65 to 55%. Our study found out that women in cohorts of total mastectomy with reconstruction were more pleased with their sexuality than women without reconstruction. The result of this quality-of-life measure falls in agreement with the study conducted by Archangelo *et al*.^26^ which analyzed that women who underwent mastectomy plus reconstruction were functioning better in terms of sexuality than women with mastectomy alone. However, Parker *et al*.^27^ in their study indicated that cancer survivors experienced a rise in sexual pleasure as time passes with no relation to surgical intervention received.

Surgical decisions are mostly based upon predictions of future well-being however in case of complex surgeries like mastectomy with reconstruction, physical functioning, and cosmetic outcome after implant both played a vital role in patient’s thinking process and often lead to an over expectation of future well-being^28^. This over estimation may result in regret or disappointment with their postoperative results. The reason could be lack of awareness or improper perioperative counselling where one might be unable distinguish between aesthetic breast augmentation surgery with oncological reconstructive surgery and assume higher baseline appearance after the procedure^28^–^30^. The decision of reconstruction after mastectomy is highly preference sensitive and various reasons are considered in decision making such as age, cultural and social background, marital status and extra cost for the procedure^28^. Patients assumption of appearance clothed and unclothed also highly influences their choice of going for reconstructive surgery after mastectomy, although patients seems to have negative opinions of unclothed appearance after reconstruction compare to clothed appearance, this may be the result of how media portray clothed appearance of women after reconstruction especially celebrities, but overall our study showed women who had reconstruction of breast were more satisfied with their sexual life comparing to other two groups^31^. We used Breast-Q questionnaire that is surgery specific for all three surgeries with different modules filled by each respective group.

There were some limitations in our study; first there is no defined time duration from surgery to measure quality of life outcomes and needs further research. The outcome of cancer free state outweighs the patient satisfaction and quality of life measures although the age of the patient can be a potential bias here, where the patients that are younger tend to be more conscious about their physical appearance and outcome comparing to women of older age and that could lead a new path for further exploration where age adjusted quality of life measures can have impact on patients satisfaction with breast, physical, psychosocial and sexual health of patients. Despite all these, this is the first study reporting quality of life domains especially sexual health of breast cancer survivors in our oriental population. This can play a role in preoperative counselling of patients for better procedure selection.

## Conclusion

Postmastectomy immediate reconstruction has a positive impact on psychosocial and sexual health than total mastectomy alone; however, conserved breast cases has better cosmetic scores. Physical well-being found similar in all three groups of surgical interventions. More research projects should be carried out in this regard with inclusion of all patients in public and private sector hospitals of rural and urban areas to better understand which factors compel patients to attain highest standards of quality of life after curative surgery.

## Ethical approval

Ethical exemption was obtained from the Institution’s Ethical Review Committee (reference number: 2022-5988-21634).

## Consent

Written informed consent was obtained from all patients prior to the interviews and copy of the written consent is available for review by the Editor-in-Chief of this journal on request.

## Source of funding

No funding was received to perform this study.

## Conflicts of interest disclosure

The authors have no conflicts of interest to declare.

## Data statement

The datasets used and/or analyzed during the current study are available from the corresponding author on reasonable request.

## Provenance and peer review

Not commissioned, externally peer-reviewed.
